# Development of an LC-MS/MS method for astaxanthin quantification in shrimp tissues and its application to detect astaxanthin variations during ovary development stages

**DOI:** 10.3389/fnut.2025.1586625

**Published:** 2025-06-20

**Authors:** Shuo Diao, Guanrong Feng, Yixuan Wang, Jingming Ma, Zhihua Lv, Mingming Yu, Yue Sun

**Affiliations:** ^1^Ocean University of China, Qingdao, China; ^2^School of Medicine and Pharmacy, Ocean University of China, Qingdao, China; ^3^MOE Key Laboratory of Marine Genetics and Breeding, College of Marine Life Sciences, Ocean University of China, Qingdao, China; ^4^Haide College, Ocean University of China, Qingdao, China

**Keywords:** astaxanthin, LC-MS/MS, quantitative analysis, tissue distribution, ovarian development

## Abstract

**Objective:**

In this study, a highly selective and sensitive LC-MS/MS method was developed to comprehensively analyze the distribution of astaxanthin across various tissues of *Litopenaeus vannamei*, as well as its variations in the hepatopancreas and ovary throughout ovarian development.

**Methods:**

The separation was performed on a BEH C8 column (1.7 μm, 2.1 × 50 mm) using a a gradient elution. The initial mobile phase composition was 0.1% formic acid in 3 mM ammonium acetate in water (solvent A) and methanol (solvent B) at a ratio of 15:85 (v/v), with a flow rate of 0.20 mL/min.

**Results:**

The assay demonstrated linearity over a concentration range of 20 to 10,000 ng/mL, with accuracy varying from −0.1 to 1.7% and precision within 2.1%. Using the established methodology, astaxanthin concentrations were quantitatively analyzed and comparatively assessed across various tissues of *L. vannamei*. Analytical results demonstrated that the ventral nerve cord and hepatopancreas exhibited the highest astaxanthin concentrations among all examined tissues, with values of 351 μg/g and 116 μg/g, respectively. During the ovarian developmental stages, astaxanthin was predominantly sequestered in the hepatopancreas during early phases, with concentrations ranging from 21.3 μg/g to 29.1 μg/g, representing a 43- to 59-fold increase compared to ovarian concentrations. However, a significant redistribution of astaxanthin was observed during the post-developmental stage, characterized by a substantial decrease to 5.74 μg/g in the hepatopancreas, concomitant with an increase to 7.47 μg/g in ovarian tissue.

**Conclusion:**

This validated LC-MS/MS method effectively quantified astaxanthin in various tissues of *Litopenaeus vannamei*, providing new insights into its tissue-specific distribution and potential role during ovarian development.

## 1 Introduction

Astaxanthin (3,3′-dihydroxy-*β*,β’-carotene-4,4′-dione) is a naturally occurring pigment found extensively in various aquatic species, including salmon, shrimp, and crab (1, 2). Astaxanthin is also a natural antioxidant with multiple pharmacological activities (3). Due to its high antioxidant capacity, astaxanthin can effectively eliminate free radicals that cause disability, disease, and death in the body. It promotes skin and brain health, helps prevent cancer, and inhibits the development of complications from diabetes (4–6). In addition, astaxanthin also possesses anti-lipid peroxidation activity (7, 8) anti-inflammatory (9, 10), anti-diabetic (11, 12) and anticancer activity (13). Related reports and studies indicate that astaxanthin can protect living organisms from diseases and plays a role in the treatment and prevention of peripheral and central nervous system diseases (14, 15).

The benefits of astaxanthin for crustaceans include greater pigmentation, enhanced growth, higher survival rates, stronger stress resistance, and improved reproductive potential (16–19). The Pacific white shrimp (*Litopenaeus vannamei*) is an economically important aquaculture animal, accounting for more than 50% of the crustacean production globally. In the shrimp farming industry, astaxanthin has been used as a feed supplement to enhance the overall coloring (20). Astaxanthin supplementation during broodstock rearing can effectively improve the quality of mature ovary in female broodstock. As mentioned earlier, astaxanthin has antioxidant properties, which may affect ovarian development (21). Li et al. (22) suggested that astaxanthin improves the development of follicles and oocytes by enhancing their antioxidant capacity. Jia et al. (23) reported that astaxanthin significantly reduced the production of reactive species in oocytes and improved the quality of the oocyte nucleus. Qiang et al. (24) showed that female Nile tilapia fed with astaxanthin exhibited reduced oxidative stress in ovarian tissue and improved oocyte development.

A sensitive and simple bioanalytical method is crucial for understanding the distribution of astaxanthin during the ovarian development process. Multiple analytical methods have been developed to quantify the astaxanthin concentrations in previous studies. An HPLC method was established for quantifying astaxanthin in *Haematococcus pluvialis* (25). Raman spectroscopy was used to detect astaxanthin in salmon fillets (26). Holtin et al. (27) employed NMR spectroscopy and LC-(APCI)MS methods to detect astaxanthin and astaxanthin esters in the microalga *Haematococcus*. Todorović et al. (28) used HPLC-DAD and LC-QTOF-MS to determine the content of astaxanthin and its esters in *H. pluvialis*. However, the detection of astaxanthin in biological samples, such as shrimp tissues, is challenging due to the complexity of the matrix. An improved method is needed to meet the requirements for highly sensitive and specific quantitative detection of astaxanthin in shrimp tissues. LC-MS/MS was employed in this study, as it allows for rapid, high-throughput analysis, particularly in multiple reaction monitoring (MRM) mode, where it can achieve highly selective and sensitive quantitative analysis (29). Through MS/MS, it can analyze product ions derived from specific precursors, thereby enhancing the specificity of compound identification with high quantitative precision (30).

This study developed a simple, rapid, and accurate protein precipitation method for astaxanthin extraction, and coupled it with an LC-MS/MS method for quantification. Astaxanthin concentrations in various shrimp tissues were determined. Changes in its levels in the hepatopancreas and ovaries were analyzed across the early, middle, and late stages of ovarian development to investigate the potential role of astaxanthin in this process.

## 2 Experiment

### 2.1 Materials and reagents

Astaxanthin (≥98%) and β-carotene (internal standard, ≥98%) were purchased from Shanghai Aladdin Biochemical Technology Co., Ltd. (Shanghai, China) and Shanghai Yuanye Biotechnology Co., Ltd. (Shanghai, China), respectively. Methanol (LC-MS grade) was obtained from Thermo Fisher Scientific (Waltham, MA, United States). Formic acid was purchased from Dikma Technologies (Beijing, China), and ammonium acetate was supplied by Shanghai Aladdin Biochemical Technology Co., Ltd. (Shanghai, China).

### 2.2 Sample collection

The experimental animals were *L. vannamei*. Tissue samples collected included the eyestalk, stomach, ventral nerve cord, intestine, epidermis, hepatopancreas, muscle, ovary, and heart. The head, shell, and epidermis were removed, and the remaining parts were collected as the blank matrix. Ovarian and hepatopancreatic tissues were classified into pre-development, mid-development, and post-development stages based on the gonadosomatic index (GSI), defined as follows: GSI < 0.9% (pre-development), GSI = 2.7–4.0% (mid-development), and GSI > 6.0% (post-development). The gonadosomatic index was calculated as follows:


GSI=(gonad mass(g)/FBW(g))x100

### 2.3 Instruments and experiment conditions

Chromatographic analysis was performed on an UltiMate 3,000 UPLC system, and mass spectrometric detection was carried out using a TSQ Quantiva™ triple quadrupole mass spectrometer (Thermo Fisher Scientific, MA, United States) operated in electrospray ionization (ESI) mode with MRM. Separation was achieved on an ACQUITY UPLC® BEH C8 column (1.7 μm, 2.1 × 50 mm; Waters, Ireland).

The mobile phase consisted of 0.1% formic acid and 3 mM ammonium acetate in water (phase A) and methanol (phase B). The initial composition was 15:85 (v/v) of phases A: B with a flow rate of 0.20 mL/min. At 1.0 min, phase B was increased to 100% and the flow rate was raised to 0.40 mL/min. The system was re-equilibrated to the initial conditions at 3.0 min.

The MRM transitions and parameters for astaxanthin and *β*-carotene were as follows: m/z 597.385 → 147.117 [M + H]^+^ for astaxanthin and m/z 536.557 → 444.508 [M + H]^+^ for β-carotene. The collision energies (CE) were set at 34 eV and 13 eV, respectively. The ion source temperature was maintained at 450°C.

### 2.4 Sample preparation

Stock solutions of astaxanthin and the internal standard (IS, β-carotene) were prepared at a concentration of 1 mg/mL in DMSO and dichloromethane, respectively. These stock solutions were diluted with methanol to obtain working solutions at concentrations of 0.400, 1.00, 2.00, 10.0, 20.0, 100, 160, and 200 μg/mL. Working solutions were further diluted with the blank biological matrix to prepare calibration standards. Calibration curves for astaxanthin were constructed using eight concentrations ranging from 20 to 10,000 ng/mL (20, 50, 100, 500, 1,000, 5,000, 8,000, and 10,000 ng/mL).

Quality control (QC) samples were prepared at three concentration levels: 60, 500, and 7,500 ng/mL. All calibration standards and QC samples were freshly prepared before analysis.

In the present study, astaxanthin and the IS were extracted from shrimp tissue using a protein precipitation method with acetonitrile. Precisely weighed shrimp tissue (221 ± 35.1 mg) was homogenized in a 1:5 (w/v) ratio with 50% methanol in water. A 50 μL aliquot of the tissue homogenate was mixed with 50 μL of IS working solution (2 μg/mL), followed by the addition of 200 μL of acetonitrile. The mixture was vortexed for 1 min, then centrifuged at 14,000 rpm and 4°C for 5 min. The resulting supernatant was carefully transferred, and a 5 μL aliquot was injected for LC-MS/MS analysis.

### 2.5 Assay validation

The developed method was validated in accordance with the U. S. Food and Drug Administration (FDA) guidelines for bioanalytical method validation (31).

#### 2.5.1 Selectivity

The selectivity of the method was evaluated by spiking astaxanthin and the IS into blank biological samples obtained from six different sources.

#### 2.5.2 Linearity and LLOQ

The calibration curve was constructed by plotting the peak area ratio of astaxanthin to the IS on the y-axis against the nominal concentrations of astaxanthin on the *x*-axis using weighted (1/*x*^2^) linear regression. The curve included eight concentration levels ranging from 20 to 10,000 ng/mL. The lower limit of quantification (LLOQ) was established based on a signal-to-noise ratio (S/N) of 10:1 relative to the baseline noise.

#### 2.5.3 Accuracy and precision

The precision and accuracy of the method were evaluated at four concentration levels: the LLOQ and three QC levels (low, medium, and high). Intra-day (within-run) precision and accuracy were assessed by analyzing six replicates (*n* = 6) of each concentration on the same day, while inter-day (between-run) precision and accuracy were evaluated over three consecutive days.

#### 2.5.4 Recovery and matrix effect

The extraction recovery of astaxanthin from tissues was evaluated by comparing the areas of spiked tissue samples at three concentration levels (*n* = 6) to those of unextracted standard samples (100%). The matrix effect of astaxanthin was assessed by analyzing QC samples at two different concentrations in post-extraction blank biological matrix.

#### 2.5.5 Stability

The stability of astaxanthin in shrimp tissue was evaluated at two concentration levels under various storage conditions. Freeze–thaw stability was assessed by subjecting six replicates of QC samples to three freeze–thaw cycles, alternating between −80°C and room temperature. Benchtop stability was determined by leaving QC samples at room temperature for over 4 h. Post-preparative stability was evaluated by storing extracted QC samples in the autosampler at 4°C for more than 15 h. Long-term stability was assessed by storing QC samples at −80°C for 1 month.

### 2.6 Statistical analysis

In this experiment, three biological replicates with three technical replicates were performed (*n* = 3 for each group). One-way analysis of variance (ANOVA) with Tukey’s *post hoc* test was used to evaluate the differences in astaxanthin concentrations among different organs.

## 3 Results

### 3.1 Method development and optimization

**Figure 1:**
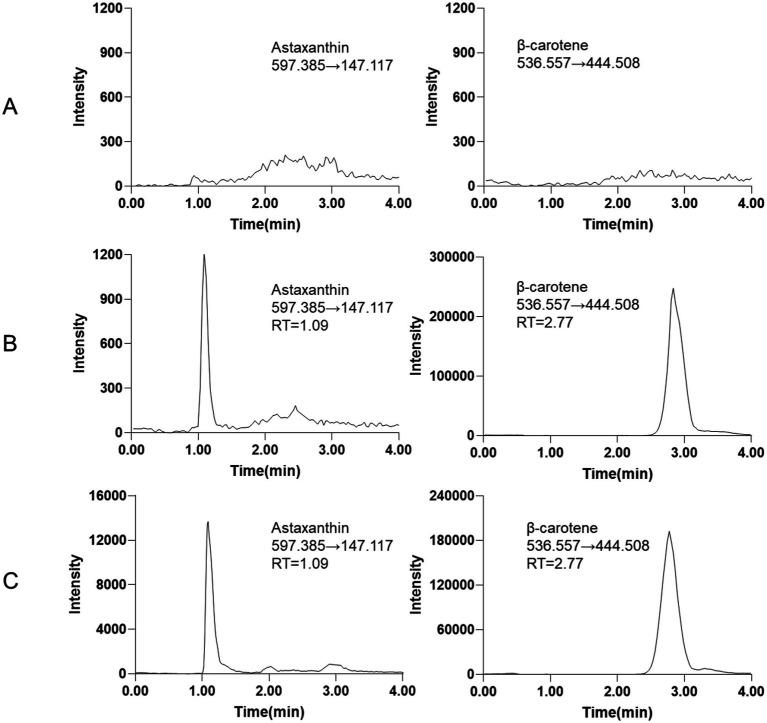
Representative MRM chromatograms **(A)** blank tissue sample. **(B)** Blank tissue spiked with LLOQ of astaxanthin (20 ng/mL) and IS (2000 ng/mL). **(C)** The epidermis tissue sample of *L. vannamei*.

In this experiment, both positive and negative ion modes were evaluated for the detection of astaxanthin and the IS. The ion abundances of both compounds were significantly higher in the positive ion mode compared to the negative mode. Therefore, the positive ion mode was selected for subsequent analyses. The optimal MRM transitions for astaxanthin and the IS were determined using a full scan approach. The selected transitions were *m/z* 597.385 → 147.117 [M + H]^+^ for astaxanthin and *m/z* 536.557 → 444.508 [M + H]^+^ for the IS. The full-scan mass spectra of astaxanthin and the IS are presented in Supplementary Figure S1. Chromatographic separation was achieved using methanol as the organic phase and an aqueous solution containing 0.1% formic acid and 3 mM ammonium acetate as the aqueous phase. Satisfactory peak shapes and high sensitivity (LLOQ = 20 ng/mL) were obtained at a flow rate ranging from 0.20 to 0.40 mL/min (Figures 1A–C).

### 3.2 Method validation

#### 3.2.1 Selectivity

The results from six different sources of blank biological samples indicated that no endogenous interference was observed at the retention times of astaxanthin and the IS, demonstrating the high selectivity of the method. Representative chromatograms are shown in Figure 1A.

#### 3.2.2 Linearity and LLOQ

The standard calibration curve for astaxanthin ranged from 20 to 10,000 ng/mL, exhibiting excellent linearity in the biological matrix with a correlation coefficient (*R*^2^ ≥ 0.999). The LLOQ was established at 20 ng/mL, as shown in Table 1. At the LLOQ level, the measured concentration of astaxanthin in biological samples was 20.1 ± 0.4 ng/mL (Supplementary Table S1).

**Table 1:** The intra-day and inter-day precision and accuracy data for the quantification of astaxanthin.

Nominal concentration (ng/mL)	Intra-Day (*n* = 6)			Inter-Day (*n* = 18)		
	Mean ± SD (ng/mL)	Accuracy (RE %)	Precision (RSD %)	Mean ± SD (ng/mL)	Accuracy (RE %)	Precision (RSD %)
20	20.1 ± 0.4	0.6	2.1	20.2 ± 0.1	1.1	0.5
60	60.3 ± 0.2	0.6	0.4	61.0 ± 0.9	1.7	1.5
500	504 ± 2	0.9	0.3	507 ± 5	1.3	0.9
7,500	7,493 ± 31	−0.1	0.4	7,511 ± 16	0.2	0.2

RE, relative error. RSD, relative standard deviation. SD, standard deviation.

#### 3.2.3 Accuracy and precision

The intra-day and inter-day accuracy and precision of the method were evaluated at four concentration levels of astaxanthin (20, 60, 500, and 7,500 ng/mL) in biological samples (Table 1). The method demonstrated excellent performance, with relative errors (RE) ranging from −0.1 to 1.7% and relative standard deviations (RSD) below 2.1%. All results were within the ±15% acceptance criteria specified by the FDA guidelines, confirming the method’s accuracy, precision, and reproducibility.

#### 3.2.4 Recovery and matrix effect

The extraction recovery of astaxanthin was evaluated at three concentration levels (60, 500, and 7,500 ng/mL). The recovery ranged from 100.3 to 100.8%, with RSD between 0.5 and 0.9% (Supplementary Table S2). The matrix effect was assessed at two concentrations using the blank biological matrix. At the low concentration level (60 ng/mL), the matrix effect was 13.8%, which was higher than that at the high concentration level (7,500 ng/mL, 1.4%), but both values remained within the acceptable limits defined by the FDA guidelines. These results indicate that the extraction process effectively minimized matrix interference. To evaluate carryover, HQC (high-quality control) samples were injected, followed by blank tissue extracts. This procedure was repeated three times, and neither astaxanthin nor the IS was detected in the blank chromatograms, confirming the absence of carryover.

#### 3.2.5 Stability

The stability of astaxanthin was evaluated under four different storage conditions: short-term storage, long-term storage, freeze–thaw cycles, and post-preparative stability (Table 2). Astaxanthin samples at low (60 ng/mL) and high (7,500 ng/mL) concentrations were analyzed to assess the impact of storage conditions. Under all tested conditions, the measured concentrations ranged from 60.6 to 62.1 ng/mL and from 7,507 to 7,526 ng/mL, respectively, with RSD below 3.4%. These results indicate that astaxanthin is stable in shrimp tissues under standard laboratory handling and storage conditions.

**Table 2:** Stability of astaxanthin in blank biological matrix (*n* = 6).

Sample condition	Nominal concentration (ng/mL)	Measured (Mean ± SD)	RSD (%)
Short-term	60	61.5 ± 1.0	1.7
	7,500	7,507 ± 12	0.2
Freeze–thaw	60	60.9 ± 1.2	1.9
	7,500	7,516 ± 34	0.4
Post-preparative stability	60	60.6 ± 0.6	1.1
	7,500	7,526 ± 387	0.5
Long-term	60	62.1 ± 2.0	3.4
	7,500	7,520 ± 55	0.7

RSD, relative standard deviation. SD, standard deviation.

### 3.3 Tissue distribution of astaxanthin

**Figure 2:**
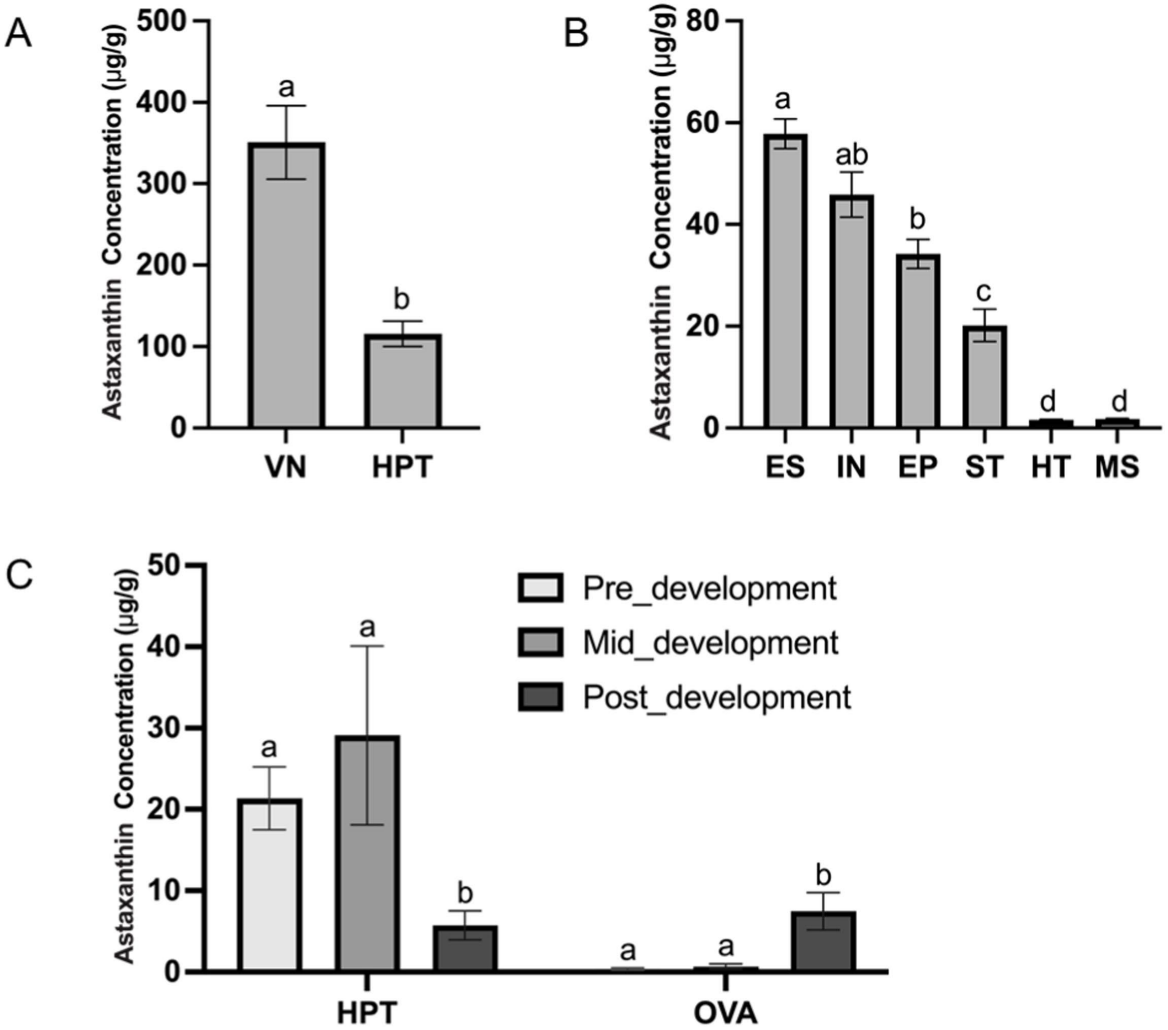
The concentrations of astaxanthin in different shrimp tissues **(A)** VN, ventral nerve cord; HPT, hepatopancreas. **(B)** ES, eyestalk; IN, intestine; EP, epidermis; ST, stomach; HT, heart; MS, muscle. **(C)** The hepatopancreas (HPT) and ovary (OVA) at different ovarian developmental stages: pre-development (GSI < 0.9%); mid-development (GSI = 2.7–4.0%); and post-development (GSI > 6.0%). Different letters (a, b, c) above bars indicate significant differences (*p* < 0.05). Bars sharing the same letter are not statistically different.

The LC-MS/MS method established in this experiment was successfully applied to detect astaxanthin concentrations in different shrimp tissues. The concentrations of astaxanthin in the eyestalk, stomach, ventral nerve cord, intestine, epidermis, hepatopancreas, muscle, and heart of *L. vannamei* are presented in Figure 2. The results demonstrated that astaxanthin was widely distributed in the nervous and digestive systems of shrimp. Among all the tissues tested, the ventral nerve cord and hepatopancreas showed the highest concentrations at 351 μg/g and 116 μg/g, respectively (Figure 2A), suggesting their critical roles for astaxanthin storage and function. The heart and muscle contained the lowest levels of astaxanthin between 1.61 and 1.76 μg/g, which was about 200-fold lower than that in the ventral nerve cord. The astaxanthin concentrations in the other organs ranged from 20.2 to 57.8 μg/g, with statistically significant differences among them based on one-way ANOVA analysis (Figure 2B). The uneven distribution of astaxanthin in different organs may be related to the physiological functions of astaxanthin in shrimp.

### 3.4 Astaxanthin dynamics during the ovarian development process

To explore the role of astaxanthin in ovarian development, we examined its concentration in the hepatopancreas and ovary, which were expected to be the major organs related to gonadal maturation in *L. vannamei*. The astaxanthin concentrations of both organs at three stages are shown in Figure 2C. The results showed that the hepatopancreas was the primary site for astaxanthin storage during early ovarian development stages with concentrations of 21.3 μg/g and 29.1 μg/g, which were 59-fold and 43-fold higher than those in the ovary, respectively. Both organs showed continuous accumulation of astaxanthin during this period. However, at the post-development stage, astaxanthin levels dropped significantly in the hepatopancreas and increased in the ovary compared with the pre- and mid-development stages. The astaxanthin concentrations eventually reached a similar level in both organs (5.74 μg/g in the hepatopancreas and 7.47 μg/g in the ovary). This suggests that the hepatopancreas may serve as an astaxanthin reservoir, storing it at early stages and transporting it to the ovary at late stage to support oogenesis.

## 4 Discussion

In this study, we developed an LC-MS/MS method to systematically analyze the distribution of astaxanthin in different tissues of *L. vannamei* and its changes in the hepatopancreas and ovary during ovarian development. Astaxanthin exists in crustaceans in both free and esterified forms (2) and is transported to various organs mainly through passive diffusion or binding to transport proteins (32). Our research showed that astaxanthin is mainly found in the nervous system, digestive system, and outer body. Many physiological processes, such as metabolism, growth, and reproduction, are regulated by the nervous system (33), which may influence the distribution and accumulation of astaxanthin by controlling the release of hormones or neurotransmitters related to its metabolism. Natural astaxanthin, produced by microalgae and phytoplankton, accumulates in zooplankton and crustaceans through the food chain (34). Since shrimp cannot synthesize astaxanthin, it must obtain it from external sources. The digestive system efficiently absorbs astaxanthin, which may bind to proteins like crustacyanin and lipoproteins (35), then transport it to various digestive organs. Additionally, the distribution of astaxanthin in the outer body may enhance stress resistance and immune function (36, 37), acting as a cellular protector and playing a key role in shrimp growth and survival.

This study also revealed dynamic changes in astaxanthin during ovarian development. As a natural antioxidant, astaxanthin protects cells from oxidative damage by scavenging free radicals (32). Combined with the hepatopancreas’s crucial role in energy metabolism and substance transport (38), these findings suggest that astaxanthin supports reproductive development by providing energy and antioxidants. As ovarian development reaches the post-development stage, astaxanthin levels in the hepatopancreas decrease, while levels in the ovary rise, supporting the hypothesis of astaxanthin transport to the ovary. This redistribution likely reflects the increased requirement for antioxidants and nutrients during late-stage oogenesis. Li et al. (22) also demonstrated that astaxanthin reduces bisphenol A-induced oxidative stress in follicles, highlighting its key role in maintaining oocyte function and ensuring ovarian development. In our research, the significant increase in ovarian astaxanthin during late-stage development suggests that oocyte growth increases the need for antioxidants. Astaxanthin supports oocyte maturation by providing antioxidant protection and maintaining organelle function, ensuring healthy ovarian development. Overall, this study contributes to a better understanding of astaxanthin’s physiological role and suggests its potential relevance in aquaculture applications, particularly in relation to reproductive performance and oocyte viability.

## Data Availability

The raw data supporting the conclusions of this article will be made available by the authors, without undue reservation.
