# Supplementary material for: Development of an LC-MS/MS method for astaxanthin quantification in shrimp tissues and its application to detect astaxanthin variations during ovary development stages
Source: Front Nutr. 2025 Jun 20;12:1586625. doi: 10.3389/fnut.2025.1586625 (PMC12226280; doi:10.3389/fnut.2025.1586625)

**Supplementary Material for**

**Development of an LC-MS/MS method for astaxanthin quantification in shrimp tissues and its application to detect astaxanthin variations during ovary development stages**

Shuo Diao^1,2^, Guanrong Feng^1,3^, Yixuan Wang^1,4^, Jingming Ma^1,4^, Zhihua Lv^1,2^, Mingming Yu^1,2^ and Yue Sun^1,3^*

1 Ocean University of China, Qingdao, China,

2 School of Medicine and Pharmacy, Ocean University of China, Qingdao, China

3 MOE Key Laboratory of Marine Genetics and Breeding, College of Marine Life Sciences, Ocean University of China, Qingdao, China

4 Haide College, Ocean University of China, Qingdao, China

*Correspondence:

Yue Sun

[sunyue@ouc.edu.cn](mailto:sunyue@ouc.edu.cn)

Corresponding Address: 5 Yushan Road, Qingdao, China, 266003

**Table S1** Analytical and statistical parameters

| Parameters |  |
| --- | --- |
| LLOQ (ng/mL) | 20 |
| Linear range (ng/mL) | 20 - 10000 |
| Linear equation | y=15251x+107.93 |
| R^2^ | 0.999 |

LLOQ, lower limit of quantitation

**Table S2** Recovery and matrix effect of the quantification of astaxanthin (n=6)

| Nominal Concentration（ng/mL） | Recovery | | Matrix Effect |
| --- | --- | --- | --- |
|  | Mean (%) | RSD (%) | RSD (%) |
| 60 | 100.8 | 0.5 | 13.8 |
| 500 | 100.7 | 0.9 | -- |
| 7500 | 100.3 | 0.5 | 1.4 |

RSD, relative standard deviation.

**Figure S1** Full product ion scans of astaxanthin (A) and the internal standard (IS, β-carotene) (B).


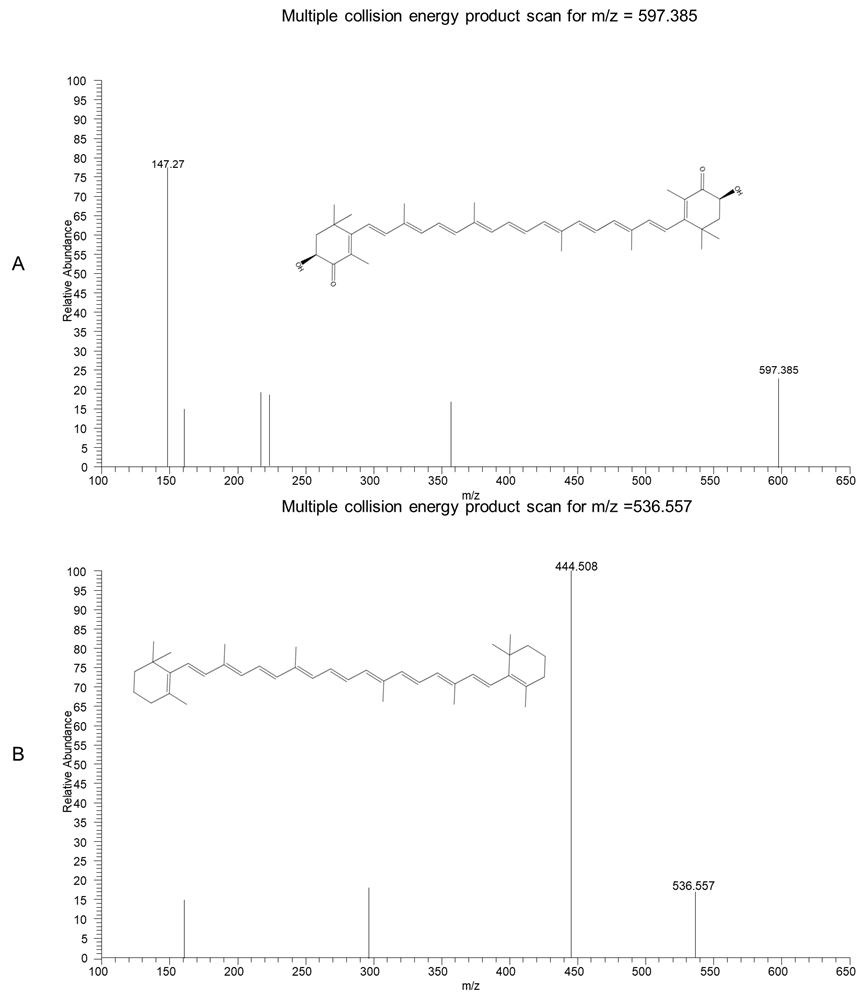

Supplement: Supplementary file 1 [file Data_Sheet_1.docx]
